# Associations of epicardial fat with coronary calcification, insulin resistance, inflammation, and fibroblast growth factor-23 in stage 3-5 chronic kidney disease

**DOI:** 10.1186/1471-2369-14-26

**Published:** 2013-01-26

**Authors:** Jasmine D Kerr, Rachel M Holden, Alexander R Morton, Robert L Nolan, Wilma M Hopman, Cynthia M Pruss, Jocelyn S Garland

**Affiliations:** 1 Department of Medicine, Queen’s University, Kingston, ON, Canada; 2 Queen’s University Vascular Calcification Investigators, Queen’s University, Kingston, ON, Canada; 3 Department of Radiology, Queen’s University, Kingston, ON, Canada; 4 Clinical Research Center, Kingston General Hospital, and Department of Community Health and Epidemiology, Queen’s University, Kingston, Ontario, Canada; 5 Department of Biomedical and Molecular Sciences, Queen’s University, Kingston, ON, Canada; 6 Room 2043 Etherington Hall, Queen’s University, Kingston, Ontario, K7L 3N6, Canada

**Keywords:** Epicardial fat, Chronic kidney disease, Coronary artery calcification, Metabolic syndrome, Interleukin-6

## Abstract

**Background:**

Epicardial fat, quantified in a single multi-slice computed tomography (MSCT) slice, is a reliable estimate of total epicardial fat volume (EFV). We sought to determine risk factors for EFV detected in a single-slice MSCT measurement (ssEFV) in pre-dialysis chronic kidney disease (CKD) patients. Our primary objective was to determine the association between ssEFV and coronary artery calcification (CAC).

**Methods:**

94 pre-dialysis stage 3–5 CKD patients underwent MSCT to measure ssEFV and CAC. ssEFV was quantified at the level of the left main coronary artery. Measures of inflammation, traditional and kidney-related cardiovascular disease risk factors were collected.

**Results:**

Mean age: 63.7 ± 14 years, 56% male, 39% had diabetes, and mean eGFR: 25.1 ± 11.9 mL/min/1.73 m^2^. Mean ssEFV was 5.03 ± 2.4 cm^3^. By univariate analysis, body mass index (BMI) (*r* = 0.53; P = <0.0001), abdominal obesity (*r* = 0.51; P < 0.0001), high density lipoprotein (HDL) cholesterol (*r* = − 0.39; P = <0.0001), insulin resistance (log homeostasis model assessment of insulin resistance (log HOMA-IR)) (r = 0.38, P = 0.001), log interleukin-6 (IL-6) (r = 0.34; P = 0.001), and log urinary albumin to creatinine ratio (UACR) (r = 0.30, P = 0.004) demonstrated the strongest associations with ssEFV. Log coronary artery calcification (log CAC score) (r = 0.28, P = 0.006), and log fibroblast growth factor-23 (log FGF-23) (r = 0.23, P = 0.03) were also correlated with ssEFV. By linear regression, log CAC score (beta =0.40; 95% confidence interval (CI), 0.01-0.80; P = 0.045), increasing levels of IL-6 (beta = 0.99; 95% CI, 0.38 – 1.61; P = 0.002), abdominal obesity (beta = 1.86; 95% CI, 0.94 - 2.8; P < 0.0001), lower HDL cholesterol (beta = −2.30; 95% CI, – 3.68 to −0.83; P = 0.002) and albuminuria (log UACR, beta = 0.81; 95% CI, 0.2 to 1.4; P = 0.01) were risk factors for increased ssEFV.

**Conclusions:**

In stage 3–5 CKD, coronary calcification and IL-6 and were predictors of ssEFV. Further studies are needed to clarify the mechanism by which epicardial fat may contribute to the pathogenesis of coronary disease, particularly in the CKD population.

## Background

The metabolic syndrome is a risk factor for type 2 diabetes mellitus [1] and cardiovascular disease (CVD) [2], and is a risk factor for the development of incident chronic kidney disease (CKD) [3,4]. This syndrome emphasizes the role of visceral abdominal adipose tissue in the pathogenesis of disease. Visceral adipose tissue is highly metabolically active and produces hormones and cytokines, including those with anti-atherosclerotic properties (e.g. adiponectin [5]) and those with pro-atherosclerotic properties (e.g. Interleukin-6 (IL-6) and tumor necrosis factor α) [6]. In obesity, the balance between such pro- and anti-atherogenic factors is disturbed. As a result, pro-atherogenic and inflammatory cytokine production increases [6], interferes with insulin signalling, and contributes to the development of insulin resistance and vascular wall inflammation [7,8].

Epicardial fat, a visceral deposit of adipose tissue located between the myocardium and visceral pericardium, is also metabolically active and produces many of the same pro-atherogenic cytokines found in visceral abdominal fat [9,10]. In the general population, epicardial adipose tissue has been shown to correlate with coronary artery calcification (CAC) [11] and the metabolic syndrome [12]. In patients with end stage kidney disease requiring dialysis, the total volume of epicardial fat is correlated with CAC [13], and is significantly greater in dialysis patients versus healthy controls [14]. CAC is an important manifestation of cardiovascular disease in CKD patients. The burden of CAC is more severe in CKD patients compared to the general population [15], in part due to the combined impact of traditional and kidney-related CVD risk factors in contributing to CAC [16]. To our knowledge, epicardial fat has not been quantified in the pre-dialysis CKD population nor its potential association with CAC or markers of inflammation.

Measurement of the total volume of epicardial fat requires radiological techniques that are time-consuming and labor-intensive in clinical settings; however, Oyama et al. determined the single-slice epicardial fat area (ssEFA), measured at the level of the left main coronary artery (LMCA), provides a reliable estimate of total epicardial fat volume (EFV) [17]. In this study, we used a similar technique as Oyama et al., and measured the single slice epicardial fat volume (ssEFV) at the level of the LMCA in a cohort of stage 3–5 CKD patients, in order to test the hypothesis that coronary calcification is more severe in CKD patients with greater epicardial fat. Therefore, our primary objective was to determine the cross sectional association between ssEFV and CAC, and our secondary objectives were to determine the associations between ssEFV and kidney function, traditional and kidney-related CVD risk factors, including markers of inflammation, obesity and insulin resistance.

## Methods

In 2005, 174 pre-dialysis CKD patients were enrolled in a study of CAC in CKD [18]. The full methods are described elsewhere, but patients were eligible to participate if they were greater than 18 years of age and had stage 3–5 CKD (not requiring dialysis and excluding acute kidney injury). All patients who had CAC scores measured in 2005 were invited to undergo repeat multi-slice CT (MSCT) scan for quantification of CAC in 2009, and had repeat clinical and biochemical assessments performed. In 2011, patients were included if they had both a CAC score result and a ssEFV result. Of the original 174 patients, 17 patients died, 31 patients progressed to dialysis, 5 were transplanted, 7 were discharged to the care of their family physician, 2 had moved, and 5 were lost to follow-up or refused consent. This left 107 patients of which 95 agreed to participate. Of these, one CAC score was not interpretable because the LMCA could not be identified due to motion artefact, leaving 94 patients who had data for both CAC and ssEFV. All patients gave informed consent, and the study protocol was approved by the Queen’s University Health Sciences and Affiliated Teaching Hospitals Research Ethics Board.

National Kidney Foundation criteria were applied to diagnose CKD [19]. Diagnoses of hypertension were documented as per 2006 Canadian Hypertension Education Program Guidelines [20], and diabetes mellitus as per the Canadian Diabetes Association criteria [21]. Metabolic syndrome was defined as fulfilling at least 3 of 5 criteria from the National Cholesterol Education Program Adult Treatment Panel III 2005 criteria [22].

### Laboratory measures

Fasting serum laboratory measured in 2009 at Kingston General Hospital’s Core Laboratory included creatinine (Jaffe rate method, Beckman Coulter UniCel DxC 800 SYNCHRON Clinical System assay, traceable to isotope dilution mass spectroscopy), glucose, phosphorus, total calcium, intact parathyroid hormone (iPTH),(chemiluminescent immunoassay, Beckman Coulter UniCel DxI 800 Access Immunoassay System, Beckman Coulter Inc., Fullerton CA), albumin, high-sensitivity C-reactive protein (hsCRP),(Beckman Coulter UniCel DxC 600/800 SYNCHRON Clinical System, Beckman Coulter Inc., Fullerton CA), total cholesterol, low density lipoprotein cholesterol (LDL), high density lipoprotein cholesterol (HDL), and triglycerides.

Blood samples were stored at –80°C, and after a single freeze-thaw cycle the following were measured from plasma in duplicate at the Ontario Cancer Biomarker Network, Toronto, ON, Canada: 1,25-Dihydroxyvitamin D, enzymeimmunoassay (Immunodiagnostic Systems Inc., Fountain Hills, Arizona), 25-hydroxyvitamin D, enzymeimmunoassay (Immunodiagnostic Systems Inc., Fountain Hills, Arizona), Fetuin A, enzyme-linked immunosorbent assay (ELISA) (ALPCO Diagnostics, Salem, NH), insulin (single measure) Human Serum Adipokine (Panel B) Kit Protocol Immunoassay, (Milliplex Analytes, Millipore Corp, St. Charles, MI), and serum FGF-23 ELISA, (measured in duplicate), (ALPCO Diagnostics, Salem, NH). Plasma IL-6 was measured in duplicate at Queen’s University using ELISA (R&D Systems, Minneapolis, MN).

The 4-variable MDRD Study equation [23], re-expressed for standardized creatinine [24], was used to estimate eGFR. Albuminuria was detected by the urinary albumin-to-creatinine ratio (UACR mg/mmol). Weight and height data were collected on each individual to calculate body mass index (BMI) in kg/m^2^. Abdominal obesity was defined as a waist circumference of >88 cm in women and >102 cm in men [22]. Insulin resistance was assessed using the following validated formula: homeostasis model assessment of insulin resistance (HOMA-IR) = (fasting glucose [mmol/1] × fasting insulin [μU/ml])/22.5 [25]. **Because of confounding introduced by exogenous insulin administration to HOMA-IR results, HOMA-IR was only measured in patients who were not treated with insulin (N = 72).**

### Coronary artery calcification measurement

CAC scores were evaluated using the General Electric (GE) VCT 64 slice helical CT scanner, (Waukesha, Wisconsin, USA) and data was processed by Smartscore software, version 3.5 from GE Medical Systems (Waukesha, Wisconsin, USA). The GE VCT 64 slice helical CT scanner scans and reconstructs 8 slices simultaneously, using a step and shoot technique. Each slice is 2.5 mm in thickness with no overlap. Images were acquired with prospective gating technique using a discrete algorithm [26]. The total CAC score was generated as per the Agatston method and reported in Agatston units (AU) [27].

### Single slice epicardial Fat volume measurement

**Figure 1:**
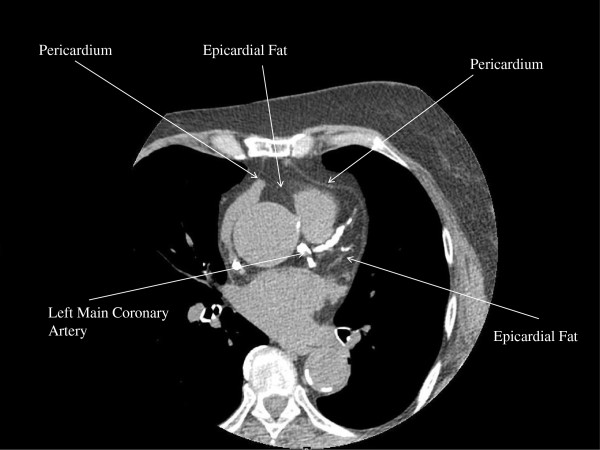
Axial MSCT scan at the left of the left main coronary artery demonstrating relevant landmarks for the measurement of single slice epicardial fat volume (ssEFV) in a 72 year old female chronic kidney disease patient.

The 2009 MSCT scans were re-analyzed in 2011 for determination of ssEFV. Oyama *et al*. [17] described a method of estimating the total EFV by MSCT scan, using a single MSCT slice located at the level of the LMCA. Single slice epicardial fat area (ssEFA) measured at the level of the LMCA correlated well with total EFV in this study (r = 0.92; P < 0.0001) [17]. We used a similar method as Oyama et al. The EFV of two MSCT slices nearest the origin of the LMCA were measured on each patient (GE Advantage Workstation, “volume tool”). Pixels in the pericardium with a density from −30 to −230 Hounsfield units were considered fat (Figure 1). The average of these two single slice epicardial fat volumes at the LMCA determined the ssEFV and was recorded in centimetres cubed. The single slice epicaridal fat area at the level of the LMCA was also measured by dividing the ssEFV by the MSCT slice thickness (2.5 mm) and recorded in centimetres squared. A random sample of 13 patients (5 patients with ssEFV 0–5 cm^3^; 4 patients with ssEFV 5–10 cm^3^, and 4 patients with ssEFV > 10 cm^3^) was chosen to verify the agreement between the two MSCT ssEFV measures. The intra-class correlation was at 0.99; 95% CI 0.96 to 1.0; P < 0.0001).

### Statistical methods

Summary statistics are expressed as means and standard deviations, medians and inter-quartile ranges (IQR), or percentages, as appropriate. Bivariate analysis was performed to evaluate the associations between ssEFV and *a priori* chosen risk factors, including CAC, BMI, abdominal obesity, HOMA-IR, hsCRP, IL-6, age, sex, hypertension, HDL cholesterol, LDL cholesterol, total cholesterol, triglycerides, diabetes mellitus, smoking status and other kidney related CVD risk factors (albuminuria (UACR), fetuin, osteoprotegerin, FGF-23, iPTH, 25- hydroxyvitamin D, and 1,25-Dihydroxyvitamin D levels. Prior to analysis, CAC, hsCRP, IL-6 and HOMA-IR were logarithmically transformed to ensure normality for parametric testing.

A series of multivariable linear regression models were constructed to identify risk factors for increased ssEFV. All variables from the bivariate analysis which showed an association with ssEFV at the significance level P < 0.10 were included in the initial multivariable linear regression models. All statistical analyses were performed using IBM SPSS Statistics 19.0.

## Results

Table 1 describes baseline clinical characteristics of the 94 included study participants. The prevalence of hypertension and diabetes were high, 32% of patients had stage 3 CKD, 47% stage 4 CKD, 19% stage 5 CKD, and 42% had evidence of macro-albuminuria (UACR > 30 mg/mmol). In general, patients did not have hyperphosphatemia but vitamin D levels were low and FGF-23 levels were elevated. 79% of patients had CAC scores greater than 10 AU (minimal CAC), and 38% having CAC scores greater than 400 AU (severe CAC).

**Table 1:** Baseline clinical characteristics of study participants (N = 94)

**Variable**	**Prevalence**	**Mean/Median**	**SD/IQR**
Age (years)		63.7	14
Male sex	56%		
Body mass index (kg/m^2^)		31.7	9.3
eGFR (ml/min/1.73 m^2^)		25.1	11.9
UACR (mg/mmol) (N = 88)		22.2	4-118
Hypertension (N = 93)	94.7%		
Systolic Blood Pressure (mmHg) (N = 93)		131.8	17.4
Diastolic Blood Pressure (mmHg) (N = 93)		73.3	11.8
Diabetes mellitus	39%		
Metabolic Syndrome	81%		
HOMA-IR (uU/mL) (N = 72)		2.19	1.19–3.94
Glucose (mmol/l)		5.7	5.2–6.7
Hemoglobin A1C% (N = 93)		6.1	5.6–7.2
Calcium (mmol/l)		2.30	.022
Phosphorus (mmol/l)		1.29	0.27
Albumin (g/l)		37.3	3.6
Total cholesterol (mmol/l) (N = 93)		4.15	1.29
HDL cholesterol (mmol/l) (N = 93)		1.01	0.34
LDL cholesterol (mmol/l) (N = 92)		2.37	1.05
Triglycerides (mmol/l) (N = 93)		1.8	1.06
hsCRP (mg/l) (N = 93)		3.00	1.25–6.1
25-Hydroxy Vitamin D (nmol/l)		54.67	44.9–69.1
1,25-Dihydroxyvitamin D (pmol/l)		49.6	30.8–71.5
FGF-23 (RU/ml)		150.8	73.4–305.3
Fetuin (g/L) (N = 93)		0.613	0.13
Osteoprotegerin (pmol/L)		4.09	1.86
Interleukin – 6 (pg/mL) (N = 93)		1.89	0.67–5.30
Coronary artery calcification score (AU)		192	24–873
ssEFV (cm^3^)		5.03	2.4
ssEFA (cm^2^)		20.1	9.77

eGFR = estimated glomerular filtration rate; UACR = urinary albumin to creatinine ratio; HOMA-IR = homeostasis model assessment of insulin resistance; HDL = high density lipoprotein; LDL = low density lipoprotein; hsCRP = high sensitivity C-reactive protein; FGF-23 = fibroblast growth factor −23; AU = Agatston units; ssEFV = single slice epicardial fat volume at the level of the left main coronary artery (LMCA); ssEFA = single slice epicardial fat area at the level of the LMCA; SD = standard deviation; IQR = interquartile range.

The average ssEFV measured at the level of LMCA was 5.03 ± 2.44 cm^3^; the corresponding average ssEFA was 20.1 ± 9.77 cm^2^. ssEFV was significantly higher is patients with metabolic syndrome versus those without metabolic syndrome (ssEFV 5.45 cm^3^ ± 2.3 cm^3^ versus 2.84 cm^3^ ± 1.9 cm^3^; P <0.0001), in patients with diabetes mellitus versus those without a diagnosis of diabetes, (ssEFV 5.74 cm^3^ ± 2.14 cm^3^ versus 4.52 cm^3^ ± 2.5 cm^3^; P = 0.02) and in patients who had increasing levels of insulin resistance. Patients with HOMA-IR values greater than the median had significantly higher ssEFV versus patients whose HOMA-IR values were less than the median (ssEFV 6.26 cm^3^; IQR 3.99 to 7.47 cm^3^ versus 3.39 cm^3^; IQR 1.89 to 4.66 cm^3^; P 0.0001).

Table 2 lists findings from the bivariate analysis examining associations of ssEFV, and *a priori* chosen risk factors. BMI, abdominal obesity, HDL cholesterol, triglycerides, insulin resistance (log HOMA-IR), markers of inflammation (log IL-6, log hsCRP, hypoalbuminemia and albuminuira) and log CAC score demonstrated the strongest associations with ssEFV. In terms of other kidney related CVD risk factors, log FGF-23 correlated with ssEFV (r = 0.23; P = 0.03); however, kidney function (eGFR) was not associated with ssEFV, missing statistical significance, nor was 25-hydroyxvitamin D, 1,25-Dihydroxy vitamin D, fetuin, osteoprotegerin or serum phosphorus.

**Table 2:** Correlations with epicardial fat volume (N = 94)

**Variable**	***r***	**P**
Age	0.06	0.60
Body Mass Index	0.53	<0.0001
eGFR	−0.18	0.09
log UACR (N = 88)	0.30	0.004
Systolic Blood Pressure (N = 93)	0.07	0.50
Diastolic Blood Pressure (N = 93)	−0.06	0.60
log Coronary artery calcification score	0.28	0.006
Abdominal obesity	0.51	<0.0001
Log HOMA-IR (N = 72)	0.38	0.001
Fasting glucose	0.23	0.02
Triglycerides (N = 93)	0.34	0.001
LDL cholesterol (N = 92)	−0.17	0.11
HDL cholesterol (N = 93)	−0.39	<0.0001
Log hsCRP (N = 93)	0.30	0.003
Log Interleukin - 6	0.34	0.001
Log FGF-23	0.23	0.03
Phosphorus	0.08	0.50
Albumin	−0.28	0.007
25-Hydroxy Vitamin D	−0.14	0.17
1,25-Dihydroxyvitamin D	−0.13	0.23
Fetuin-A (N = 93)	−0.13	0.20
Osteoprotegerin	0.03	0.80

eGFR = estimated glomerular filtration rate; UACR = urinary albumin to creatinine ratio; HOMA-IR = homeostasis model assessment of insulin resistance; HDL = high density lipoprotein; LDL = low density lipoprotein; hsCRP = high sensitivity C-reactive protein.

FGF-23 = fibroblast growth factor −23.

A series of multivariable linear regression models (Tables 3 and 4) were developed to determine risk factors for ssEFV, adjusted for level of kidney function and diabetes mellitus. Coronary artery calcification, increasing levels of IL-6, abdominal obesity, lower HDL cholesterol, and albuminuria were significantly associated with greater ssEFV. In a separate multivariable regression model including metabolic syndrome as a co-variate (rather than the individual metabolic syndrome components), an association between metabolic syndrome and ssEFV was observed (metabolic syndrome ß = 1.8; 95% confidence interval 0.41 to 3.2; P = 0.01)(full model not shown). This association no longer remained robust once adjusted for body mass index (ß = 1.2; 95% confidence interval −0.07–2.45; P = 0.06) (Table 4). Considering insulin resistance, although HOMA-IR was correlated with ssEFV, this association no longer remained in the multivariable regression model (N = 72; HOMA-IR ß =1.3 ; 95% confidence interval −0.47 – 3.1; P = 0.15) (full model not shown).

**Table 3:** **Multivariable regression risk factors for single slice epicardial fat volume (N = 94), R**^**2**^ **= 0.49**

**Model 1 Co-Variates**	**EFV Beta co-efficient**	**P**
	**(95% Confidence Interval)**	
Log Coronary artery calcification score	0.40 (0.01–0.80)	0.045
Abdominal obesity	1.86 (0.94–2.80)	<0.0001
Log UACR	0.81 (0.20–1.40)	0.01
HDL cholesterol	−2.30 (−3.68–-0.83)	0.002
Interleukin −6	0.99 (0.38–1.61)	0.002
Diabetes mellitus	−0.86 (−1.8–0.10)	0.08
eGFR (per 10 ml/min/1.73 m^2^ decrease)	0.25 (−0.15–0.65)	0.21

UACR = urinary albumin-to-creatinine ratio; HDL = high density lipoprotein; eGFR = estimated glomerular filtration rate.

**Table 4:** **Multivariable regression risk factors for single slice epicardial fat volume (N = 94), R**^**2**^ **= 0.49**

**Model 2 Co-Variates**	**EFV Beta co-efficient**	**P**
	**(95% Confidence Interval)**	
Log Coronary artery calcification score	0.48 (0.06–0.88)	0.025
Log UACR	0.25 (−0.44–0.97)	0.48
Interleukin −6	0.73 (0.14–1.30)	0.02
Diabetes mellitus	−0.47 (−1.38–0.45)	0.31
eGFR (per 10 ml/min/1.73 m^2^ decrease)	−0.05 (−0.45–0.35)	0.81
Body Mass Index (Kg/m2)	.015 (.09–0.21)	<.0001
Metabolic syndrome (yes/no)	1.2 (−0.07–2.45)	.06

UACR = urinary albumin-to-creatinine ratio; eGFR = estimated glomerular filtration rate.

## Discussion

Risk factors for epicardial fat have not been previously assessed in pre-dialysis CKD patients, who are recognized to have a markedly increased risk of CVD and adverse cardiovascular events, compared to individuals from the general population [28]. Our results demonstrate the following findings:

1. The burden of epicardial fat volume is greater in individuals with metabolic syndrome, increased insulin resistance or diabetes mellitus.

2. By univariate analysis, epicaridal fat volume is correlated with coronary artery calcification, body mass index, dyslipidemia, insulin resistance (HOMA-IR), abdominal obesity, albuminuria and interleukin-6. FGF-23, a biomarker indicating disrupted phosphorus homeostasis, was also correlated with EFV.

3. In the multivariable adjusted regression models, the association between epicardial fat volume and coronary artery calcification remains robust. This observation, while not causal, suggests epicardial fat deposition may be important in the etiology of CAC.

Epicardial fat has been quantified in various ways in the literature; however, measures of EFV in pre-dialysis CKD patients are not available. We employed a similar technique as Oyama *et al*. [17] to quantify EFV, which involved measuring the ssEFV and ssEFA at the level of the LMCA. While we recognize that differences in patient populations limit comparisons across studies, the average ssEFA in the pre-dialysis CKD cohort from this study population (BMI 31.7 ± 9.3 kg/m^2^, 39% diabetes, 95% hypertension), appears to be substantially greater (20.1 ± 9.77 cm^2^) than results obtained in the Oyama study (4.4 ± 2.2 cm^2^) of 72 Japanese men from the general population (BMI 23.4 ± 3.0 kg/m^2^, 38% diabetes, 60% hypertension) [17].

Our data demonstrate ssEFV was greater in CKD patients with metabolic syndrome, insulin resistance, or diabetes. Although metabolic syndrome and insulin resistance were correlated with ssEFV in univarate analyses, these associations did not remain in the multivariable regression models, with metabolic syndrome just missing statistical significance (Table 4). However, the multivariable regression model including the individual components of metabolic syndrome as covariates demonstrated abdominal obesity and low HDL cholesterol to be predictors of ssEFV (Table 3). Yerramasu *et al*. have reported similar findings in 333 diabetic (non CKD) patients, where the association between metabolic syndrome and EFV no longer remained significant in the multivariable adjusted regression model once adjusted for body mass index [11].

Epicardial adipose tissue is emerging as a novel risk factor for CVD development [29] and progression [11]. It is hypothesized that the proximity of epicardial fat to the coronary arteries, along with their shared microcirculation, allows pro-atherogenic hormones and cytokines released from epicardial adipose tissue to act locally in a paracrine or vasocrine manner to promote coronary disease [10,30]. We evaluated the association between markers of inflammation (IL-6, hsCRP, hypoalbuminemia and albuminuria) and ssEFV in this pre-dialysis CKD cohort. Of these, the associations between IL-6 (Tables 3 and 4) and albuminuria (log UACR, Table 3) and ssEFV remained robust in the multivariable adjusted models. In dialysis patients, Turkmen *et al*. also reported that epicardial fat correlated with the MIAC syndrome (malnutrition, inflammation (assessed by hsCRP), atherosclerosis/ calcification)), and that the burden of epicardial fat increased as the number of MIAC components increased [14]. Albuminuria is an important risk factor predicting the likelihood of progressive kidney function deterioration [31], is a marker of endothelial dysfunction [32], is a risk factor for CVD, and is associated with increased abdominal adiposity [33]. Taken together, these data suggest a relationship between inflammation, epicardial adipose tissue, and the development of coronary disease, in CKD patients.

The mechanism regarding IL-6 and epicardial fat remains uncertain; however, both circulating, and local IL-6 production have been demonstrated as risk factors for epicardial fat deposition in the general population. For example, Yerramasu *et al*. showed that IL-6 levels were significantly higher across increasing tertiles of epicardial adipose tissue in asymptomatic diabetic patients [11]. Shibasaki *et al*. compared IL-6 expression in the epicardial adipose tissue of patients with CAD compared to those without CAD, and showed that while IL-6 levels were increased when sampled from epicardial fat, circulating levels of IL-6 did not differ between the two groups [34].

To our knowledge, the association we report between IL-6 and ssEFV has not been previously reported in pre-dialysis CKD patients. Importantly, IL-6 has been shown to be a risk factor for CKD development. A large prospective study of 1500 patients demonstrated IL-6 was associated with the development of incident CKD [35]. The association we report between IL-6 and ssEFV remained when adjusted for kidney function (eGFR) and albuminuria (Table 4), suggesting a link between elevated circulating inflammatory markers in CKD and local inflammation in fat surrounding the coronary arteries.

Since the coronary arteries and epicardial adipose tissue share the same micro-circulation, we also sought to determine the associations between epicardial adipose tissue and coronary calcification. CAC, a form of vascular calcification described in approximately 70% of predialysis CKD patients, is increased in CKD patients compared to age and sex match individuals from the general population. The mechanism of accelerated vascular calcification in CKD reflects disequilibrium between CAC inhibitors (eg fetuin A) and promoters (eg: abnormal bone and mineral metabolism). We have reported previously that approximately 70% of pre-dialysis CKD patients demonstrate CAC, and that CAC is increased in patients with higher BMI, suggesting a link between obesity and CAC [36].

In this study, ssEFV and CAC were associated, and this finding remained robust in the multi-variable regression Model 1 adjusted for abdominal obesity, albuminuria, renal function, diabetes mellitus, HDL cholesterol, and IL-6 (Table 3). These data suggest that in addition to promoting local atherosclerosis, excess epicardial adipose tissue may also contribute to the development of vascular calcification in CKD patients.

We also evaluated the associations of ssEFV with kidney-related CVD risk factors, including markers of abnormal bone and mineral metabolism (phosphorus, iPTH, 25-hydroxyvitamin D, 1,25-Dihydroxyvitamin D, and FGF-23). Of the tested CKD bone and mineral metabolism parameters, only FGF-23 was associated with ssEVF (log FGF-23 (r = 0.23; P = 0.03); however, this association no longer remained in the multivariable regression models. FGF-23, a phosphaturic hormone secreted by osteocytes, is a biomarker indicating disrupted phosphorus homeostasis in CKD patients [37]. FGF-23 facilitates the maintenance of normal serum phosphorus levels by decreasing renal phosphorus re-absorption in the kidney proximal tubule, and consequently FGF-23 levels are elevated early in the course of CKD, and increase as kidney function declines [38].

To our knowledge, this is the first study to report an association between FGF-23 and EFV. Data supporting a potential link between FGF-23, atherosclerosis, and measures of obesity are limited. A study of 128 hemodialysis patients demonstrated FGF-23 to be correlated with carotid artery intima media thickness [39], and another study in elderly individuals with normal renal function, demonstrated FGF-23 levels to be significantly higher in subjects with increased BMI, waist circumference and total body fat mass as measured by dual x-ray absorptiometry [40]. Our results are consistent with these findings, and suggest a potential signal between excess epicardial adipose tissue and disrupted phosphorus homeostasis, but future studies are needed to explore this hypothesis.

There are limitations to this study worth consideration. First, we quantified the ssEFV at the level of the LMCA, and as such, our results do not represent the total EFV. Nevertheless, the single slice method correlates well with total EFV reported in the study by Oyama *et al*., the method is easy, to use and has been demonstrated to be reproducible [17]. Second, our sample size of 94 individuals may have been underpowered to demonstrate associations of EFV with other traditional and kidney-related CVD risk factors. Third, the optimal measures of insulin resistance are obtained by the euglycemic hyperinsulinemic clamp procedure; however, it was not possible to perform clamp procedures in this study. We wished to employ a tool for measuring insulin resistance which could be easily applied to clinical settings, and thus the use of HOMA-IR was the preferred method. In addition, since HOMA-IR was measured in 72 versus 94 patients, the lack of association between EFV and HOMA-IR may reflect inadequate sample size. Finally, our reported associations of ssEFV and measures of obesity, insulin resistance and metabolic syndrome, may have occurred because such measures are inter-related. Further studies will be required to confirm these findings and whether the role of epicardial fat in contributing to CVD differs in CKD patients versus the general population.

## Conclusions

The ultimate goal of understanding the pathogenesis of CVD in CKD patients is to discover new methods of risk stratification and interventions for prevention and treatment. The proximity of epicardial fat to the coronary arteries suggests a potentially novel biological mechanism for accelerated coronary calcification in pre-dialysis CKD patients. To date, no studies have assessed whether interventions to decrease epicardial fat improve cardiovascular, renal or metabolic outcomes in CKD patients. Further studies are needed to clarify the mechanism by which epicardial fat may contribute to the pathogenesis of coronary disease, particularly in the CKD population.

## Abbreviations

MSCT: Multi-slice computed tomography (MSCT); EFV: Epicardial fat volume; ssEFV: Single slice epicardial fat volume; CKD: Chronic kidney disease; CAC: Coronary artery calcification; BMI: Body mass index; HDL: High density lipoprotein; HOMA-IR: Homeostasis model assessment of insulin resistance; IL-6: Interleukin-6; UACR: Urinary albumin to creatinine ratio; FGF-23: Fibroblast growth factor-23; CVD: Cardiovascular disease; ssEFA: Single slice epicardial fat area; LMCA: Left main coronary artery; iPTH: Intact parathyroid hormone; hsCRP: High sensitivity C-reactive protein; LDL: Low density lipoprotein; ELISA: Enzyme-linked immunosorbent assay; IQR: Interquartile range.

## Competing interests

Drs. Garland, Holden and Morton have received paid honoraria for providing continuing medical education and consulting fees. Dr. Garland has received paid honoraria for providing continuing medical education for Amgen Canada, Eli Lilly, and Boehringer-Ingelheim. Dr. Holden has received paid honoraria from Hoffman LaRoche. Dr Morton has received paid honoraria from Genzyme Canada Inc., Novartis, and Shire Biochem Inc.

## Authors’ contributions

JG conceptualized the idea for this manuscript with significant contributions from JK, ARM and RH. WH assisted with the statistical analysis. CP assisted with the immunoassay measurements. RN interpreted the epicaridal fat volume and coronary artery calcification results. All authors contributed to the manuscript writing, and approved the final submission.

## Pre-publication history

The pre-publication history for this paper can be accessed here:

http://www.biomedcentral.com/1471-2369/14/26/prepub
